# Intraspecific scaling of home range size and its bioenergetic association

**DOI:** 10.1002/ecy.70003

**Published:** 2025-02-06

**Authors:** Evan E. Byrnes, Jenna L. Hounslow, Vital Heim, Clemency E. White, Matthew J. Smukall, Stephen J. Beatty, Adrian C. Gleiss

**Affiliations:** ^1^ Centre for Sustainable Aquatic Ecosystems Harry Butler Institute, Murdoch University Murdoch Western Australia Australia; ^2^ Environmental and Conservation Sciences Murdoch University Murdoch Western Australia Australia; ^3^ Department of Environmental Sciences, Zoology University of Basel Basel Switzerland; ^4^ Faculty of Health and Life Sciences University of Exeter Exeter UK; ^5^ Bimini Biological Field Station Foundation South Bimini Bahamas; ^6^ Present address: Oceans Department, Doerr School of Sustainability Stanford University Stanford California USA

**Keywords:** acceleration, allometry, body mass, elasmobranch, fish, home range, metabolic rate, ontogeny, scaling, space use

## Abstract

Home range size and metabolic rate of animals are theorized to scale in relation to body mass with similar exponents. This expectation has only been indirectly tested using lab‐derived estimates of basal metabolic rate as proxies for field energy requirements. Therefore, it is unclear if existing theory aligns with observed patterns of home range scaling since field metabolic rates may scale differently than basal metabolic rates. We conducted the first direct field test of the relationship between home range and metabolic rate allometry. Using acoustic telemetry, we simultaneously measured the home range sizes and field metabolic rates of lemon sharks (*Negaprion brevirostris*) spanning one order of magnitude in body mass and compared the allometric scaling exponents of these traits. Similarity between allometric scaling exponents confirmed an expected strong association between metabolic rate and home range size. However, a nonsignificant but negative association between standard metabolic rate (SMR) and home range size suggests a complex relationship between metabolism and home range, contrasting previous assumptions of a positive relationship. Nevertheless, an overall positive association between home range size and total metabolic rate persisted, driven by a strong association between active energy expenditure and home range size. These findings underscore the intricate relationship between energetics and home range size, emphasizing the need for additional direct field investigations and the potential for modern tagging technologies to gather relevant data.

## INTRODUCTION

With the exception of nomads and migrants, animals tend to move within a restricted home range, the size of which is widely observed to increase allometrically with body size (Teitelbaum & Mueller, 2019). The size of home ranges has long been thought to scale proportionately with the metabolic rate of animals, as their home range must provide access to sufficient resources for sustenance. To test this idea, McNab (1963) estimated the allometric scaling exponents (i.e., slopes) of interspecific mammalian basal metabolic rate (BMR) and home range size and found both scaled with a similar exponent. This finding led to the assertion that home range size is directly proportional to metabolic requirements. Subsequent field studies, however, found that home range size scaled at substantially greater exponents than predicted by McNab's hypothesis (e.g., Lindstedt et al., 1986; Minns, 1995; Pearce et al., 2013). The most prominent explanation for such discrepancies is that animals with overlapping home ranges share resources, and as animals grow, the proportion of shared resources and the costs of defending them increases (Damuth, 1981; Jetz et al., 2004). Contrary to the assumptions of this explanation, not all species actively defend territories or resources; some share resources through exploitative competition (Case & Gilpin, 1974). Accordingly, field studies have presented conflicting support for this shared resource hypothesis (e.g., Ofstad et al., 2016; Pearce et al., 2013).

One potential, yet rarely acknowledged, explanation for home range size scaling with a higher exponent than predicted by existing theory is that the field metabolic rates (FMRs) of animals scale more steeply than assumed in current models. Various methodological constraints have limited our ability to estimate metabolic rate over long time scales (see Butler et al., 2004; Wilson et al., 2020), making it previously impossible to directly compare the allometries of FMR and home range (noted by McNab, 1963). As a result, current hypotheses on the relationship between metabolic rate and home range size use estimates of animals' BMR (Haskell et al., 2002; Jetz et al., 2004; McNab, 1963), operating under the implicit assumption that BMR and FMR scale with similar exponents. However, the FMR of animals includes costs associated with foraging and evading predators, growth, and in mature individuals, reproduction, all of which may respond to different ecological circumstances. Therefore, animals require more resources and, consequently, more space than predicted based on BMR scaling exponents alone. Empirical data across various taxa have shown that FMR and other measures of metabolic rate including activity (e.g., routine, active, and maximal) scale with allometrically higher exponents than maintenance (i.e., basal or standard) metabolic rates (Glazier, 2008, 2009, 2010; Nagy, 1987; Weibel et al., 2004). Therefore, observations where home range size has scaled with greater exponents than predicted by McNab (1963) may simply be a product of animals attempting to acquire additional resources to meet the demands of their FMR.

Clarifying the relationship between metabolic rate and home range size has been challenging because a gamut of factors other than metabolism are also associated with home range size. These factors include an organism's biological characteristics, such as locomotory mode and speed, thermoregulatory strategy, foraging niche and dimensionality (Tamburello et al., 2015), sex‐related behavioral differences (Dhellemmes et al., 2023), and social organization and interactions (Papageorgiou & Farine, 2020). In addition, home range size may be influenced by environmental characteristics, such as resource distribution and habitat productivity (Gompper & Gittleman, 1991; Walton et al., 2017), and ecological characteristics, such as predation risk (Ofstad et al., 2016). Despite extensive research on the mechanisms associated with home range size, particularly bioenergetics, the absence of FMR data has hindered the ability to assess its impact on home range size.

Disentangling the various drivers of home range size is particularly difficult in higher vertebrates. In birds and mammals, parental investment and complex social systems impact metabolic demands (Alonso‐Alvarez & Velando, 2012; Pearce et al., 2013), confounding the metabolic association of individual home range size across different life stages. However, in many lower vertebrates these mechanisms can be more easily separated. For example, elasmobranchs are a useful model group because they grow by several orders of magnitude during development and are self‐sufficient foragers throughout all life stages. This enables us to investigate home range allometry independent of variation in metabolic demands owing to different life histories and biological characteristics between taxa.

In this study, we address two key questions about the spatial ecology of animals by concurrently quantifying home range size and FMR of lemon sharks (*Negaprion brevirostris*). Importantly, we produce the first estimates of FMR across an order of magnitude of body mass using acceleration as a metabolic proxy (Gleiss et al., 2011; Wilson et al., 2006), which we comprehensively validated previously across the same scales of body mass (Byrnes et al., 2021). First, we apply this novel dataset to test whether the scaling of home range size and FMR conform to predictions made by existing theory, by comparing the scaling exponents of both traits. Second, we take advantage of the simultaneous collection of movement and bioenergetic data to gain a causal understanding of how energy expenditure contributes to variation in home range size using structural equation modeling.

## METHODS

### Study site and species

The study was conducted at Bimini, Bahamas (25°44′ N, 79°16′ W), a mangrove‐fringed chain of islands located approximately 85 km east of Miami, Florida, USA. The Bimini Islands enclose an approximately 21‐km^2^ lagoon that is between 0 and 1.2‐m deep at low tide. The relatively shallow water depth limits the abundance of large marine predators, providing a nursery habitat for juvenile lemon sharks (Heupel et al., 2007; Morrissey & Gruber, 1993). Individuals show high site fidelity to their pupping area through at least the first three years of life (Morrissey & Gruber, 1993), after which home range size increases and sharks gradually disperse into deeper and less‐protected habitats around the lagoon, with emigration out of the lagoon near sexual maturity (~1.7‐m precaudal length, Brown & Gruber, 1988). This high site fidelity through the early life stages allows for reliable quantification of home range size and daily metabolic requirements over long periods of time for individuals spanning a continuum of body sizes. Additionally, focusing on juveniles allowed us to preclude the influence of reproduction on spatial behaviors (e.g., migrations) or energy costs (e.g., somatic growth).

### Tagging and data preparation

From April 2019 to August 2019, 20 lemon sharks were captured in the lagoon between North and South Bimini using a combination of handline, drumline, and long‐line fishing methods. Upon capture, sharks were sexed, measured for length (precaudal [PCL], fork [FL], and total [TL]) and implanted with an acoustic tag (V13AP; Vemco, Innovasea, NS, Canada). Tags were surgically implanted in the peritoneal cavity of each shark through a 4‐cm incision that was sealed with two simple interrupted sutures using poliglecaprone 25 sutures (Q310 MonoWeb, Patterson Veterinary, Devens, MA, USA). As part of other studies, a fin clip, muscle biopsy, and blood sample were also taken from each shark prior to release. This entire sampling and tagging procedure lasted between 10 and 15 min.

**FIGURE 1:**
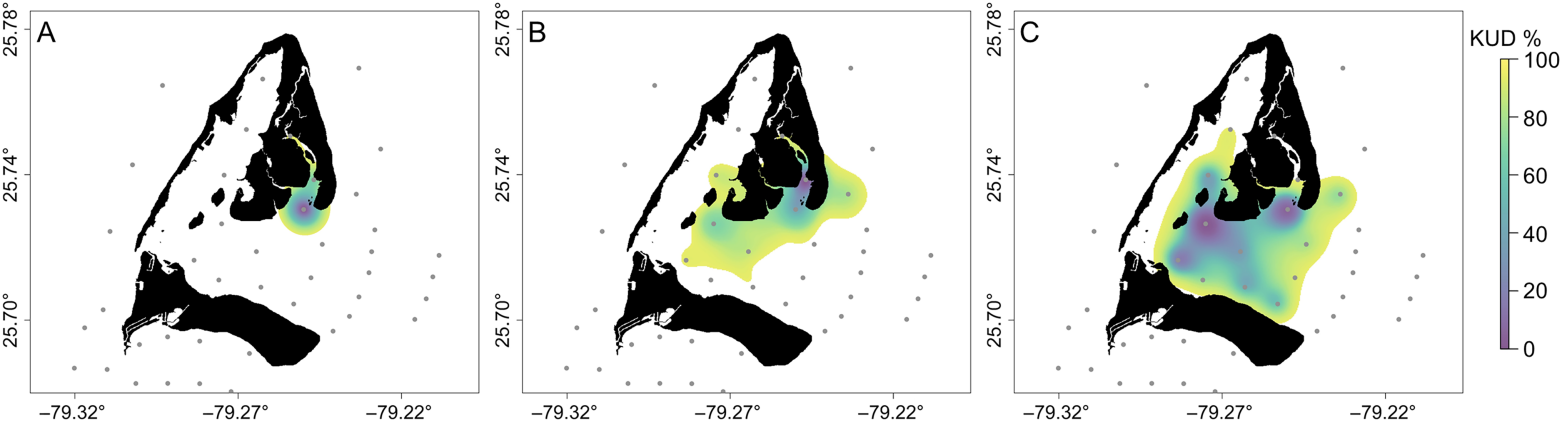
Home ranges of three lemon sharks (*Negaprion brevirostris*), in Bimini, BHS, demonstrating how home range size increased with body size. For clarity, only three sharks are displayed, representing the smallest (left; 2.32 kg and 1.82 km^2^), a midsized (middle; 9.74 kg and 9.38 km^2^), and the largest (right; 17.76 kg and 16.71 km^2^) individuals. Receiver locations are indicated by gray dots; not all 60 receivers included in figure. Home ranges for all tagged individuals provided in Appendix S1: Table S1. Kernel Utilisation Distribution (KUD) probabilities indiacted by color.

After release at the site of capture, tags alternately transmitted body acceleration (in meters per second squared) and depth (in meters) data at a nominal delay of 90–180 s. Body acceleration data were recorded at 5 Hz for a period of 20 s, which was processed onboard tags as mean vectorial dynamic body acceleration (VeDBA) over the entire recording duration. Tag transmissions were recorded by a network of 60 underwater receivers (VR2W; Vemco, Innovasea, NS, Canada) placed around Bimini (Figure 1). Temperature loggers (HOBO U22‐001 and HOBO MX2201; Onset Computer Corp., MA, USA) were attached to 40 receivers, representing all available habitats, and recorded ambient water temperature every 10 min.

In December 2019 and January 2020, receivers and temperature loggers were retrieved to download acoustic detections and temperature data. Prior to analyses, data of the first week for all tags were removed to allow sharks to recover from the tagging procedure, and all double and false detections were removed from the dataset as per Kessel et al. (2014). To ensure that home range and metabolic rate estimations were consistent over the same time frame for all sharks, only data from when all tags were active were used for analysis (July 28th through December 12th, 2019). All animal use was conducted in accordance with permits from the Bahamas Department of Marine Resources (MA&MR/FIS/178) and Murdoch University Animal Ethics committee (RW3119/19).

### Home range analysis

Home range size (95% utilization distribution) was calculated by fitting Brownian bridge movement models (BBMMs) to acoustic data for each individual across the study period using functions provided in the “Animal Tracking Toolbox” extension of the “VTrack” package (Udyawer et al., 2018) in R (version 3.6.3, R Core Team, 2019). For preparation of home range analysis, three‐hourly mean geographic positions (i.e., centres‐of‐activity) were estimated for each shark using the “COA” function. The use of mean geographic position estimates in home range analysis, rather than raw locations, provides a more accurate representation of animal movement by accounting for temporally variable tag transmissions and spatial biases from fixed receiver locations (Simpfendorfer et al., 2002).

BBMMs were applied to centre‐of‐activity estimates from each shark using the “HRsummary” function. BBMMs require input of two initial parameters: (1) the Brownian motion variance (σ^2^_m_), representing how diffusive or irregular the movement of an animal is, and (2) the error associated with location estimates. The σ^2^_m_ was estimated within the “HRsummary” function with the minimum number of independent locations adjusted to three (Kranstauber et al., 2012). Location error was set to 255 m, which is equal to the detection range estimated for receivers within our study site (Guttridge et al., 2017). Land was excluded from utilization distributions by overlaying them on a habitat raster. Areas overlapping land were manually clipped, and the remaining utilization distribution was estimated to the nearest 1 m^2^.

### Metabolic rate estimation

To estimate the daily energy demand of each shark, mean daily FMR was back‐calculated using the bioenergetic equation

(1) FMR=Production+Expenditure+Excretion.

Production was calculated based on the estimated growth rate of each shark. Growth rate was estimated using a von Bertalanffy growth curve established for lemon sharks (Brown & Gruber, 1988):

(2) $$\mathrm{PCL}=317.65\times \left(1-e^{-0.057\left(t+2.302\right)}\right),$$

where PCL is precaudal length at the time of capture and *t* is the estimated age of the fish at time of capture. The age of a shark upon capture was estimated by inserting the precaudal length at capture into Equation (2). Then one year was added to the age at capture, and a presumptive precaudal length was estimated using Equation (2). Mass of the shark at time of capture and one year later were estimated based on exponential precaudal length to weight relationships established from sharks captured at Bimini as part of other studies ($\text{Weight}=0.23\times e^{0.04\times \mathrm{PCL}}$; *r* ^2^ = 0.91, *n* = 382, unpublished data). The difference between these masses was converted to a daily energy equivalent by multiplying it by the energy content of lemon shark tissue (5.4 kJ g^−1^, Cortes & Gruber, 1994) and dividing the result by 365 days.

Expenditure was estimated using daily mean oxygen uptake rates (*Ṁ*O_2_) as a proxy. *Ṁ*O_2_ was predicted for each acoustic detection using the oxygen uptake rate predictive equation established for this population of lemon sharks by Byrnes et al. (2021):

(3) $$ṀO_{2}=\left(\left(433.87\times M^{1.55}\right)\times \left(\text{VeDBA}\right)\right)+\left(154.51\times M^{1.08}\right),$$

where VeDBA is tag‐derived measurements of acceleration in *g* and *M* is an individual's body mass in kilograms. Importantly, this equation was validated to remove body‐size‐associated estimation bias, enabling its application across individuals varying in body size (Byrnes et al., 2021). Prior to incorporation into Equation (3), VeDBA values were corrected to account for acceleration sensor noise; sensors at complete rest record small acceleration values that can inherently inflate VeDBA measurements. Raw VeDBA observations were categorized as either active (i.e., swimming) or inactive (i.e., resting) using histogram segregation, with higher values indicating active and lower values indicating inactive behavior (Appendix S1: Figure S1; Collins et al., 2015). Individual sensor noise was estimated as the mean VeDBA recorded during inactive behavior and subsequently subtracted from all active VeDBA observations. Inactive VeDBA observations were all set to equal zero, as these should represent periods when animals were resting motionless on the bottom.

To account for the effect of temperature on *Ṁ*O_2_, the intercept of Equation (3) was adjusted using a *Q*_10_ relationship (Clarke, 2017):

(4) $$Q_{10}={\left(\frac{R_{2}}{R_{1}}\right)}^{\frac{10}{T_{2}-T_{1}}}\;\text{or}\;R_{2}=R_{1}\times {Q_{10}}^{\frac{T_{2}-T_{1}}{10}},$$

where *Q*_10_ is the temperature correction factor of *Ṁ*O_2_, *T*_1_ is the temperature at which Equation (3) was calibrated (29.50°C), *T*_2_ is the observed water temperature at the time of a respective detection, *R*_1_ is the *Ṁ*O_2_ estimated at *T*_1_, *R*_2_ is the *Ṁ*O_2_ at *T*_2_. *Q*_10_ values of 2.96 for inactive detections and 1.69 for active detections were applied for temperature adjustments (Lear et al., 2017).

Water temperatures used in *Ṁ*O_2_ predictive equations were estimated using random forest (RF) regression models (Appendix S1). Hourly water temperature was estimated at each receiver location based on a suite of environmental variables (Appendix S1: Table S1), which were then time‐matched to acoustic detections. Overall, RF models for each receiver had a mean squared error ranging from 0.17 to 1.41°C (mean: 0.67°C; Appendix S1: Table S2).

Lastly, excretion was assumed to equal 27% of FMR, based on an 80% absorption efficiency (Wetherbee & Gruber, 1993) and 7% loss of assimilated energy in gill and urine effluent (Brett & Groves, 1979).

To assess how the inactive and active portions of FMR covaried with home range size, we also estimated the standard metabolic rate (SMR; analogous to BMR for homeotherms) and portion of metabolic rate due to exercise (hereafter called EMR). SMR, defined here as the resting metabolic rate of an ectothermic animal (Brett & Groves, 1979), was estimated by calculating the daily FMR using Equation (3), with VeDBA set to zero. EMR was estimated by subtracting SMR from FMR for each individual.

### Statistical analysis

To explore the association between home range size and metabolic rates (FMR, SMR, and EMR), two main types of analyses were conducted using R (version 3.6.2; R Core Team, 2019). Statistical significance for all analyses was determined based on CI overlap and using an alpha ≤0.05.

First, we compared the allometric scaling exponents of home range size and metabolic rate using least squares regression in the R stats package. Before fitting regressions, home range, FMR, SMR, EMR, and body mass were natural‐log (i.e., log_e_) transformed. Ln‐transformed home range size, FMR, SMR, and EMR were then separately regressed against ln‐transformed body mass to determine allometric scaling exponents. Differences in scaling exponents were determined based on overlap of CIs, calculated using the confint function. Model formulas were exponentiated to establish the power functions describing the relationship between body mass and the home range size, FMR, SMR, and EMR.

**FIGURE 2:**
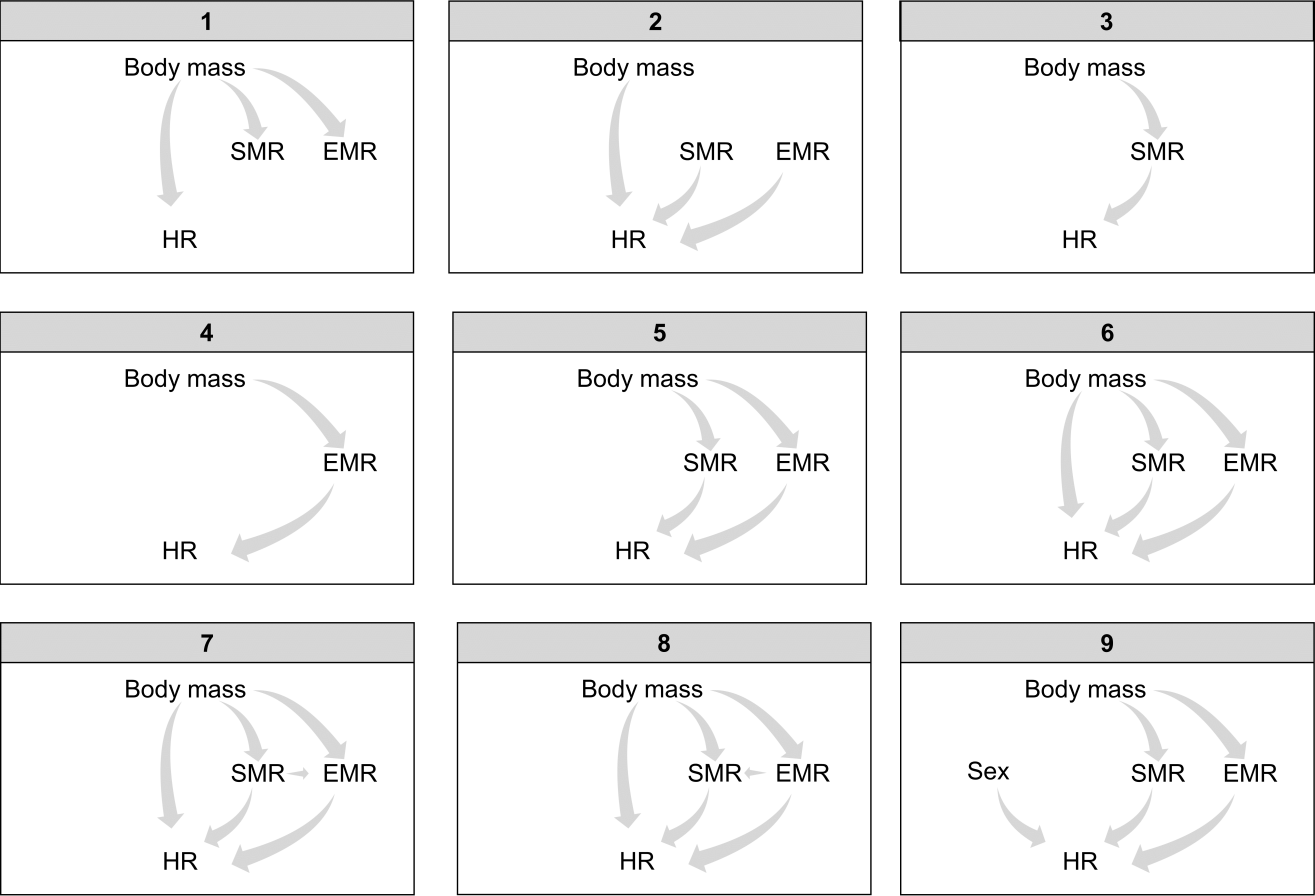
Nine candidate models used in the confirmatory path analysis. EMR, exercise‐related metabolic rate; HR, home range size; SMR, standard metabolic rate.

Second, to directly quantify the association between home range size and metabolic rates, we conducted linear modeling of the allometric scaling residuals, along with a confirmatory path analysis (i.e., structural equation model). To quantify how home range size and metabolic rate covaried while controlling for body mass effects, we regressed the residuals from the allometric relationships—specifically the residual home range size against residual SMR and residual EMR. Due to potential biases associated with analysis of residuals (Freckleton, 2002), we also conducted path analyses using the lavaan package to confirm relationships (version 0.6.17; Rosseel, 2012). We tested if the association of metabolic rate with home range size was mediated by body mass and if SMR, EMR, and sex predicted home range size using nine candidate models (Figure 2). Candidate models were evaluated using Akaike information criterion corrected for small sample size (AIC_c_; Hurvich & Tsai, 1989), and the best‐fit model was selected as the model that minimized AIC_c_. Model goodness‐of‐fit was evaluated based on the chi‐square test (χ^2^/df; Schumacker & Lomax, 2010), root mean square error of approximation (RMSEA), the comparative fit index (CFI), and Tucker–Lewis index (TLI; Hu & Bentler, 1999). Significance of the standardized regression coefficients of the best‐fit model was tested with 1000 bootstrap replicates generated within the cfa function of the lavaan package (version 0.6.17; Rosseel, 2012).

## RESULTS

### Tag deployments

Two of the 20 tagged individuals were only detected within the first 12 days after tagging and were assumed to have left the receiver array, providing insufficient data to estimate home range size. Home range size and daily FMR were estimated for the remaining 18 individuals, which ranged in estimated body mass from 2.32 to 17.76 kg (Appendix S1: Table S3). One of these 18 sharks was also excluded from further analysis because it demonstrated an uncharacteristically small home range, indicating that it died or shed its tag (Appendix S1: Table S3). For the remaining 17 individuals, home range size ranged from 2.04 to 21.35 km^2^; overall daily FMR ranged from 712.83 to 7358.18 kJ day^−1^, and SMR from 374.68 to 3382.10 kJ day^−1^ (Appendix S1: Table S3). Sharks were active for 93.40% to 100.00% of detections. Overall, temperatures experienced by sharks ranged from 20.20 to 41.40°C. Mean temperatures experienced varied among individuals, ranging from 26.10 to 30.10°C (Appendix S1: Table S3). However, there was no evidence that mean temperature experienced was associated with body mass (Appendix S1: Figure S2).

### Home range size and metabolic rate allometry

**FIGURE 3:**
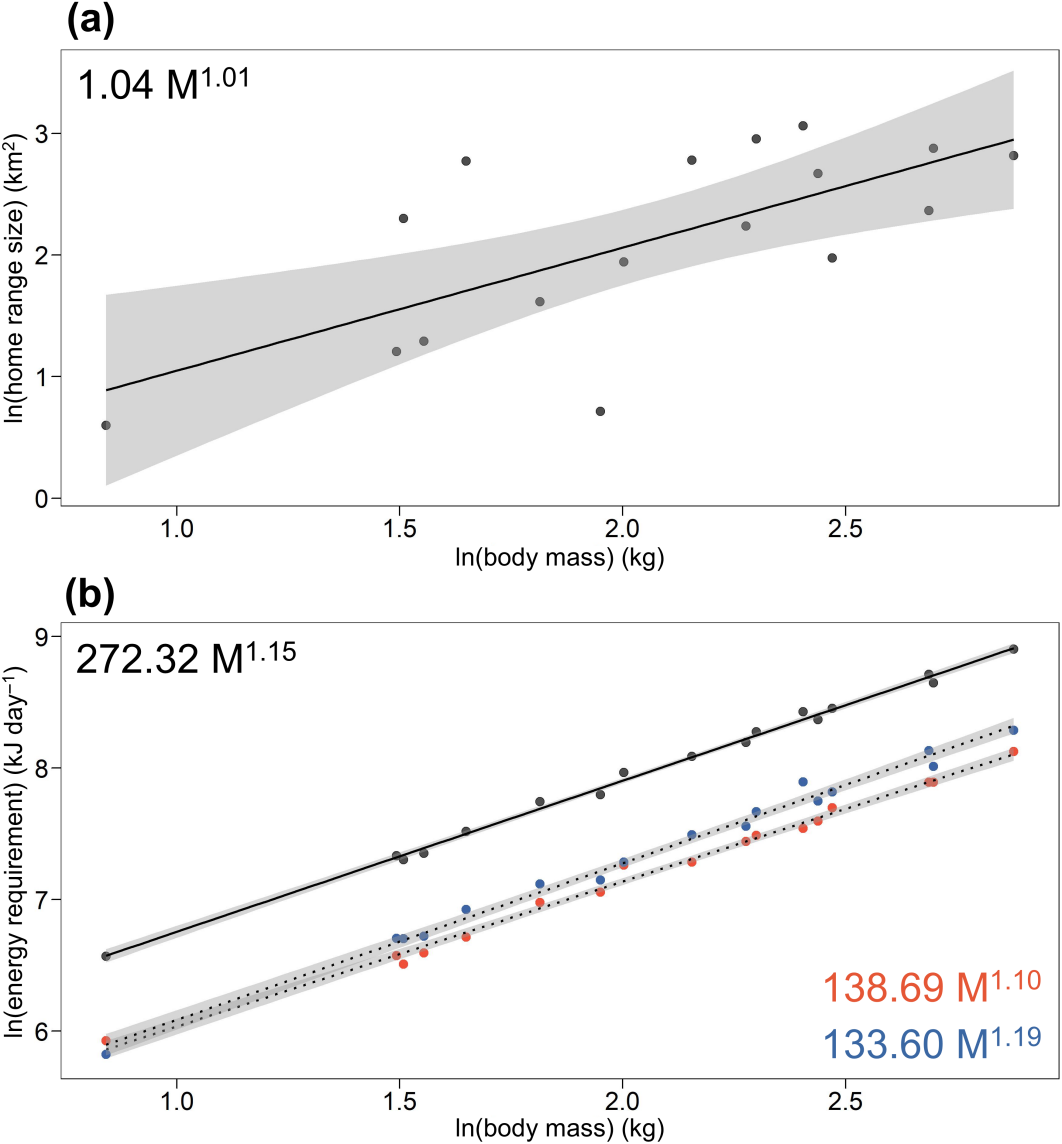
Allometric power scaling (natural‐log linear) relationship of (a) home range size and (b) metabolic rates. Scaling of daily field metabolic rate (FMR, black points), daily standard metabolic rate (SMR, blue points), and daily exercise‐related metabolic rate (EMR, red points) are plotted together for comparison. Power scaling relationships are shown for each relationship, established by exponentiation of the natural‐log relationship. Dashed lines represent power scaling relationships estimated for components of FMR (SMR and EMR) to visually distinguish them from the FMR. Scaling intercepts and slope provided in Table 1. Shaded area shows 95% CI.

Confirming previous studies, home range and metabolic rate had similar positive allometry. Home range size scaled with an allometric exponent of 1.01 (Table 1, Figure 3a), whereas FMR scaled with an exponent of 1.15 (Table 1, Figure 3b). However, the estimates of inactive (SMR) and active (EMR) portions of metabolic rate scaled with significantly different allometric exponents: SMR scaled with an exponent of 1.10, while EMR scaled with an exponent of 1.19 (Table 1).

**TABLE 1:** Comparison of power scaling (natural‐log linear) relationship intercepts and slopes.

Metric	Intercept	Slope	*r* ^2^
Home range	0.04 (−1.12 to −1.19)	1.01 (0.47 to 1.55)	0.46
Field metabolic rate (FMR)	5.61 (5.53 to 5.68)	1.14 (1.11 to 1.18)	1.00
Standard metabolic rate (SMR)	4.93 (4.85 to 5.02)	1.10 (1.06 to 1.14)	0.99
Exercise‐related metabolic rate (EMR)	4.89 (4.79 to 5.00)	1.19 (1.14 to 1.24)	0.99

*Note*: CIs presented in parentheses.

### Home range size and metabolic rate covariation

**FIGURE 4:**
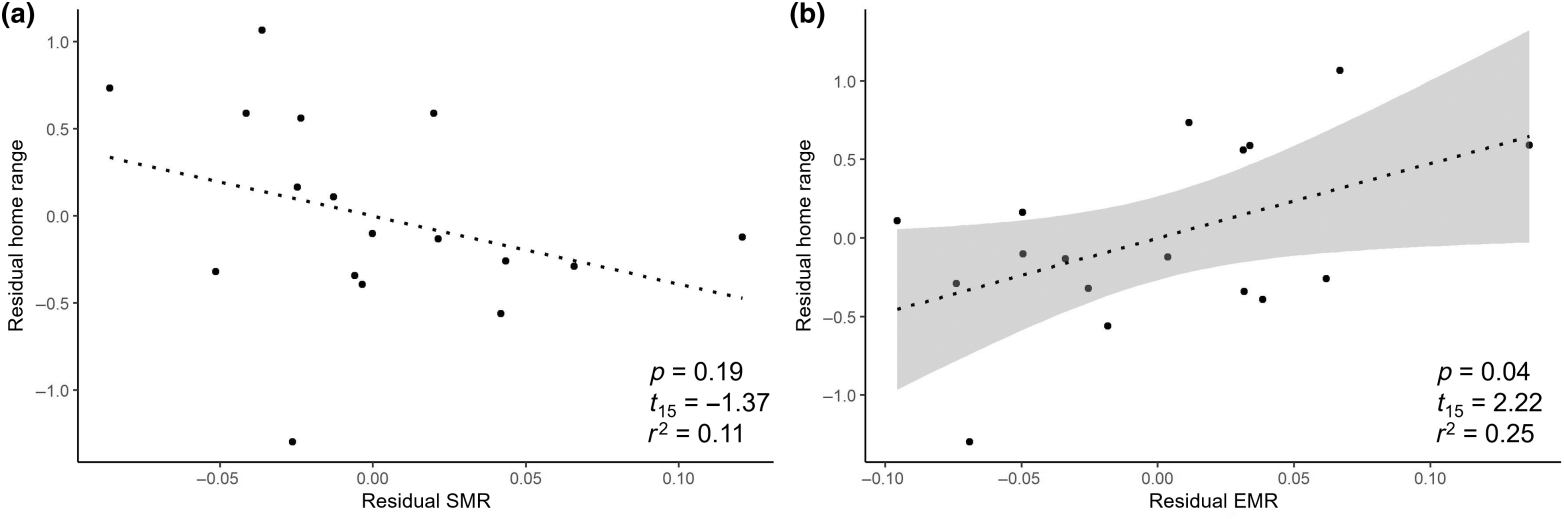
Residuals of home range size plotted against residuals of (a) standard metabolic rate (SMR) and (b) EMR (i.e., exercise‐related metabolic rate). These residuals were calculated from least squares linear regression of the natural‐log of each variable with the natural‐log of body mass. The simple linear regression lines, coefficient of determination *r* ^2^, test statistic with df, and *p*‐value are presented in each plot. Shaded area shows the 95% CI for the significant association.

After accounting for the effect of body mass, residual EMR was positively associated with residual home range size, while residual SMR showed a negative, though nonsignificant, covariation with home range size (Figure 4).

**FIGURE 5:**
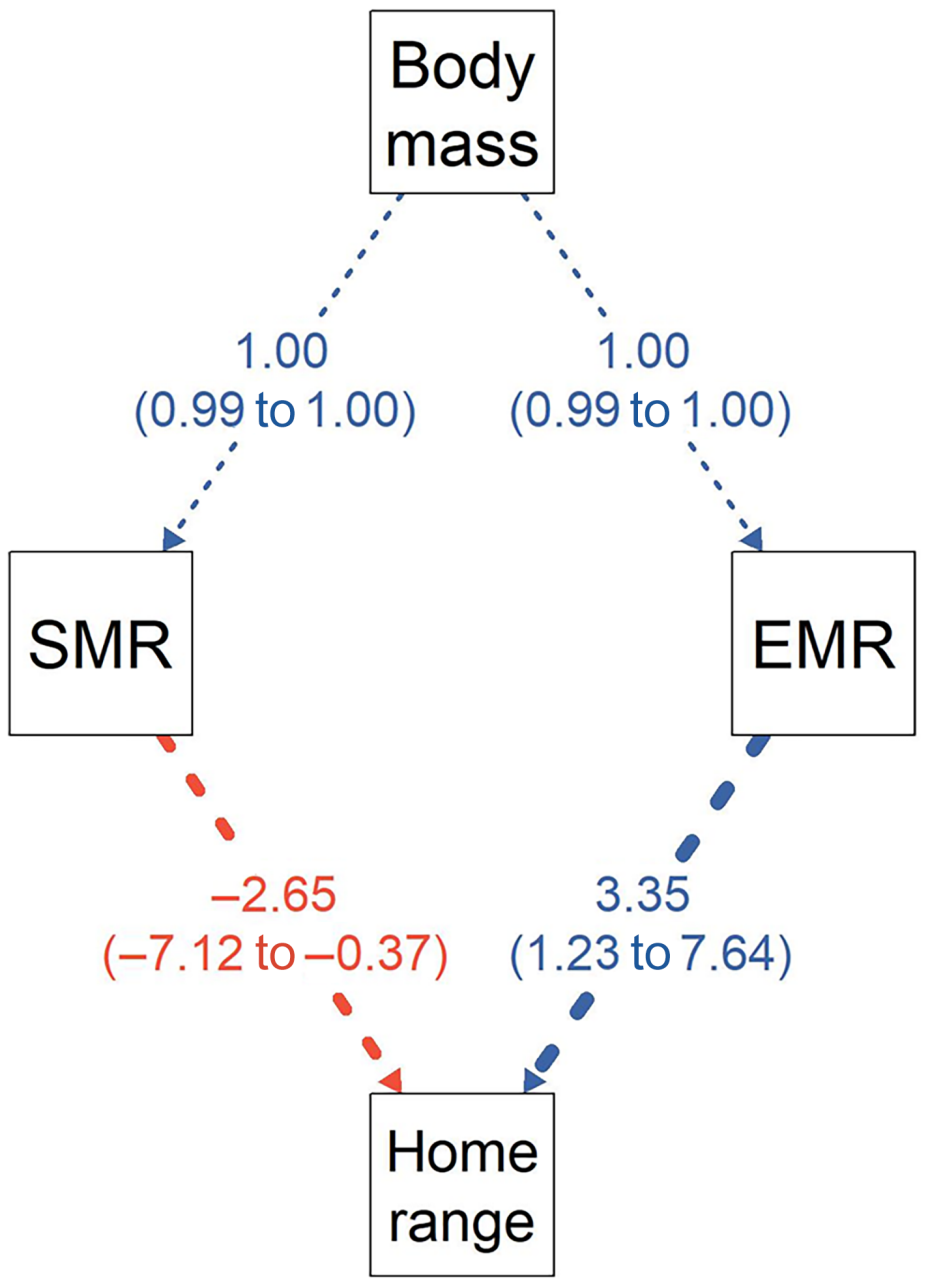
Visual representation of the best‐fitting structural equation model describing the association between biological predictors and home range size. Arrows reflect positive (blue) and negative (red) paths with line thickness proportional to their standardized regression coefficients (presented along with bootstrapped 95% CI). EMR, exercise‐related metabolic rate; SMR, standard metabolic rate.

Path analysis showed similar results: a nonsignificant negative path between SMR and home range size (standardized coeff = −3.49; Figure 5) and a significant positive path between EMR and home range size (standardized coeff = 4.09; Figure 5). Model selection suggested the inclusion of the pathway between SMR and home range size provided the best‐fit model (Table 2), and despite its marginal significance, we decided to retain SMR in the final model. The standardized effect of EMR (3.35) was larger than that of SMR (−2.65; Figure 5). Further, body mass was indirectly associated with home range via significant positive paths with SMR and EMR (Figure 5).

**TABLE 2:** Model selection table for path analysis examining the relative association of metabolism, body mass, and sex with home range size.

M_ID_	Formula	df	AIC_c_	ΔAIC_c_	χ^2^/df	CFI	TLI	RMSEA	SRMR
1	HR ~ Mass SMR ~ Mass EMR ~ Mass	9	−38.29	16.84	0.00	**1.00**	**1.00**	**0.00**	0.00
2	HR ~ Mass + SMR + EMR	4	33.29	88.43	0.00	**1.00**	**1.00**	**0.00**	0.00
3	HR ~ SMR SMR ~ Mass	4	−14.56	40.57	**0.09**	**0.98**	0.94	0.34	0.01
4	HR ~ Act EMR ~ Mass	4	−10.03	45.10	**0.07**	**0.98**	0.92	0.37	0.01
5	HR ~ SMR + EMR SMR ~ Mass EMR ~ Mass	7	−55.13	0.00	**0.81**	**1.00**	**1.03**	**0.00**	0.01
6	HR ~ Mass + SMR + EMR SMR ~ Mass EMR ~ Mass	8	−47.62	7.51	**0.53**	**1.00**	**1.02**	**0.00**	0.01
7	HR ~ Mass + Act SMR ~ Mass EMR ~ Mass + SMR	9	−46.38	8.75	**0.20**	**1.00**	**0.98**	0.19	0.01
8	HR ~ Mass + SMR SMR ~ Mass + Act EMR ~ Mass	9	−43.56	11.58	0.04	**0.98**	0.88	0.45	0.01
9	HR ~ SMR + EMR + Sex SMR ~ Mass EMR ~ Mass	8	−49.59	5.55	**0.92**	**1.00**	**1.04**	**0.00**	0.01

*Note*: Mass = body mass in kilograms; sex = male or female. Models are shown using R's notation, *Y* ~ *X*. Models were compared using corrected Akaike information criterion (AIC_c_), and model fit to data were assessed using chi‐square test (χ^2^/df), comparative fit index (CFI), Tucker–Lewis index (TLI), root mean square error of approximation (RMSEA), and standardized root mean square residual (SRMR). Values of model fit indices that met the recommended threshold are bolded (χ^2^/df > 0.05; Schumacker & Lomax, 2010; CFI > 0.95, TLI > 0.95, RMSEA < 0.06; Hu & Bentler, 1999). M_ID_ corresponds to model numbers presented in Figure 2. M_ID_ 5 is the best‐fit model.

Abbreviations: EMR, exercise‐related metabolic rate; FMR, field metabolic rate; HRS, home range size; SMR, standard metabolic rate; VeDBA, daily vectorial dynamic body acceleration.

## DISCUSSION

Home range size is associated with energetics (Tamburello et al., 2015) and is expected to increase proportionally to increased daily energy expenditure (McNab, 1963), but field data validating this relationship have been absent. Here, we provide the first field‐derived evidence confirming a primary association of energetics with home range size. By combining measurements of FMR and home range size from individuals spanning one order of magnitude in body mass, we confirm similarity between the allometric scaling exponents of home range and total energy expenditure (i.e., daily FMR). The positive allometric scaling of both traits implies a positive association between home range size and energy expenditure, consistent with theories relating metabolism and home range scaling (Damuth, 1981; Gittleman & Harvey, 1982; McNab, 1963). However, our nuanced investigation of metabolic component‐specific relationships with home range size reveals divergent associations with different constituents of total energy expenditure.

The negative (though nonsignificant) association between SMR and home range observed here suggests a positive relationship may not hold across all types of metabolism. A similar, but significant, relationship was previously reported in mammals (Boratyński, 2020; Enriquez‐Urzelai & Boratyński, 2022); the weaker relationship herein may be attributed to our small sample size (*N* = 17). Nevertheless, consistency across taxa suggests that higher levels of maintenance metabolism may be linked with decreased home range size, potentially via two non‐mutually exclusive mechanisms. First, the aerobic scope of animals has been shown to negatively associated with maintenance metabolism (Norin et al., 2016) or independent of it (Auer et al., 2015). Consequently, higher maintenance costs can constrain the amount of energy available to be allocated to locomotion, a concept known as the metabolic compensation hypothesis (Nilsson, 2002), thereby functionally limiting home range size (Boratyński, 2020). Second, under high levels of resource competition, animals are expected to use larger home ranges due to limited resource availability (Jetz et al., 2004). However, high levels of resource competition concurrently select for more conservative pace‐of‐life strategies, as lower maintenance metabolic demand decreases the risk of starvation when resources are limited (Wilson, 2014). In support of this hypothesis, previous studies showed that slower‐growing juvenile lemon sharks have higher survival rates, indicating selection for lower maintenance costs operates within our study system (Dibattista et al., 2007). This selection has been partially attributed to high levels of resource competition caused by annual influxes of juvenile sharks each pupping season (Dibattista et al., 2007; Gruber et al., 2001). While we cannot determine the influence of either mechanism here, experiments manipulating competitor densities across ecologically similar habitats could help clarify how these mechanisms underlie negative associations between maintenance metabolic rate and individual home range size.

The direction of causation between home range size and metabolic rate has long been debated. Metabolic rate may drive home range size due to the need to travel more widely to obtain sufficient food to support a higher metabolic rate (McNab, 1963). Alternatively, home range size may drive metabolic rate because greater travel costs increase metabolic rate (Glazier, 2015). Our finding that the significant relationship between home range size and metabolic rate was specific to the active component of metabolic rate (i.e., EMR) supports the latter hypothesis.

It is possible that the observed associations between metabolic rate and home range were confounded by constraints imposed by other factors that influence the movements of animals, including predator avoidance (Lima & Dill, 1990), social pressure (Bode et al., 2011), and physiological performance (Huey, 1991; Whitlock et al., 2015), among others (Shaw, 2016). Disentangling the effects of these variables is complicated by spatial and temporal variability in the relative influence of these factors, which is affected by motivations of animals associated with changes in endogenous and exogenous conditions (Sih, 1980). Additionally, the motivation of animals may differ among individuals due to variations in personality traits (Nilsson et al., 2014). While we were unable to disentangle the relative effects of such confounding factors, future quantification of daily movement distances and energy expenditure in relation to foraging events could offer insights into whether variation in motivation to acquire energy resources prompts animals to expand their home range area.

Cross‐taxonomic observations of positive allometry in both home range size and maintenance metabolic rate have reinforced the ecological principle of their positive relationship (Tamburello et al., 2015). However, our direct quantification of metabolic rate and home range size contradicts this principle, highlighting a major pitfall in drawing conclusions about the directionality of relationships between variables based solely on allometric scaling comparisons. Analysis of the relationships between scaling residuals yielded similar results to the path analysis and could provide a simple, yet valuable addition to future scaling analyses for confirming the direction of relationships.

The association between metabolism and home range size has repeatedly been investigated indirectly via scaling exponents due to the unavailability of FMR data. However, modern multi‐sensor tagging technologies now allow for the simultaneous measurement of both movement and metabolic proxies, thus enabling direct quantification of the relationship between home range size and bioenergetics. Furthermore, analyses of biologging sensor data have been developed to estimate the quantity and frequency of energy intake in some animals (e.g., Sato et al., 2008; Whitlock et al., 2015), increasing capabilities to model the metabolic dynamics of free‐ranging animals. Employing tag‐derived proxies to estimate FMR rates of animals involves inherent imperfections, necessitating meticulous calibration to mitigate and comprehend estimation errors and their impact on conclusions (Wilson et al., 2020). Nonetheless, we urge the field of home range scaling to harness these new approaches to more directly assess the mechanisms underpinning variation in home range size. Combined with systematic studies of home range size across populations representing a continuum of ecological conditions (e.g., forage density and predator density), these contemporary technologies will enable a more comprehensive understanding of the interaction between bioenergetic and home range scaling across populations, ecosystems, and taxa.

## CONFLICT OF INTEREST STATEMENT

The authors declare no conflicts of interest.

## Supporting information


Appendix S1.

## Data Availability

Data (Byrnes et al., 2023) are available on Figshare at https://doi.org/10.6084/m9.figshare.22285531.v2.
