# Supplementary material for: Intraspecific scaling of home range size and its bioenergetic association
Source: Ecology. 2025 Feb 6;106(2):e70003. doi: 10.1002/ecy.70003 (PMC11800060; doi:10.1002/ecy.70003)
Supplement: Supplementary file 1 — Appendix S1. [file ECY-106-e70003-s001.pdf]

APPENDIX S1

accompanying the article:
Intraspecific scaling of home range size and its bioenergetic association

Evan E. Byrnes*, Jenna Hounslow, Vital Heim, Clemency White, Matthew Smukall, Stephen J. Beatty, Adrian C. Gleiss

*Corresponding author: eebyrnes530@gmail.com

Ecology

ELECTRONIC APPENDIX

Table of contents

Page(s)

Supplementary methods: fitting of Random Forest models	2-3
Table S1: Variables used in RF models	4
Table S2: RF model performance	5
Table S3: Metadata table for all tagged sharks	6
Figure S1: Histogram segregation plots	7
Figure S2: Temperatures experienced by individual sharks	8
Figure S3: Variable importance for RF model	9
Appendix References	10

Supplementary methods: fitting of Random Forest models

Multiple temperature loggers flooded or failed prematurely, so temperature data was either missing or was incomplete for many receiver locations. To reduce data incompleteness, we estimated water temperature using RF regression models, which has been shown to be an accurate method for environmental time-series forecasting *(Naing and Htike 2015)*. A separate RF model was built for each receiver location where recorded water temperature data was available (n=16). For receivers with no available recorded water temperature data, temperatures were assumed to be the same as the closest receiver with similar habitat type and depth.

A random forest (RF) predict approach takes the aggregate prediction of multiple regression trees *(Breiman 2001)*. Each tree in the forest is created from a bootstrapped sample of training data for the total number of trees (ntree). The number of branches at each tree (mtry) is selected from a random subset of the input variables (p). New data is predicted by taking an average prediction of ntree regression trees. An internal estimate of error rate, mean square error (MSE), is calculated by predicting the data not contained at each bootstrap sample (out-of-bag, OOB; 36% of input data) and aggregating the OOB predictions.

All random forest regression analysis was performed in R (version 3.5.2). Models were trained using a dataset consisting of all observations of water temperature recorded by the respective temperature logger (°C) as the target (i.e., response) variable and a suite of corresponding environmental input (i.e., predictor) variables (Table S1). Where water temperature was unknown, the target variable did not contain data. RF models cannot predict from unordered categorical (factor) input variables. One-hot-encoding converts an unordered categorical vector to multiple binarised vectors where each binary vector of 1 and 0 indicates the

presence of a class of the of the original vector. We used the one_hot function in the package mltools *(version 0.3.5; Gorman 2018)* one-hot encode tide phase.

Random forests were grown on the training dataset using the package randomForest *(version 4.6.14; Liaw and Wiener 2002)*. We set mtry = 6 (default mtry = p/3) and ntree = 1000. For each receiver location, we predicted unknown water temperatures (°C) by fitting the trained model using the predict function in base R. We then assessed mean square error (MSE) as a measure of predictive performance for each RF regression model at each receiver location. RF models also allow for assessment of input variables importance for further interpretation, based on random variable selection for growing the RF (Fig. S3, S4, S5, S6). For each receiver location, variable importance was ranked by %IncMSE, indicating the increase of the MSE when the given input variable is randomly permuted. Overall, RF models for each receiver had a mean squared error ranging from 0.17 - 1.41 °C (mean: 0.67 °C; Table S2). All acoustic detections were matched with water temperature based on the temporally closest available water temperature for the respective receiver.

Table S1. Variables used for random forest regression models to predict unknown water temperature for each receiver location.

Variable type	Variable	Unit	Source
target	Water temperature	°C	1
input	Day of the year		1
	Hour of the day		1
	Air temperature	°C	2
	wind direction	° (0-360)	2
	Precipitation	mm	2
	Cloud cover	%	2
	Barometric pressure	bar	2
	Sun angle	° (from horizon)	3
	Tide phase	High, Low, Rising High, Rising Low, Falling High, Falling Low	4
	Sea surface temperature	°C	5
	Lunar illumination	%	6

Note: Data sources: 1) in-situ temperature loggers; 2) www.worldweatheronline.com;
3) *oce* package in R (Kelley and Richards 2017); 4) www.tide.mobilegeographics.com; 5) www.seatemperature.info;
6) *lunar* package in R (Lazaridis 2014)

Table S2. Random forest regression model performance for each receiver location, indicated by out-of-bag mean-squared error (OOB-MSE).

REC #	OOB MSE
1	0.80
2	1.61
3	0.28
4	0.27
5	1.41
6	0.43
7	0.17
8	0.26
9	0.18
10	1.07
11	0.33
12	0.72
13	0.46
14	1.20
15	1.09
16	0.37

Table S3. Summary of biometrics, home range size, field metabolic rate (FMR), standard metabolic rate (SMR), and energy expenditure due to activity (EMR) of all sharks.

Date Tagged	Sex	Total length (cm)	Mass (kg)	No. of detections	% detections active	No. days detected	Mean temp (°C)	Home range size (km^2)	Mean FMR (kJ day^{-1})	Mean SMR (kJ day^{-1})	Mean EMR (kJ day^{-1})
19/05/2019	M	78	2.32	822	100	105	29	1.82	712.83	374.68	338.15
16/05/2019	M	97	4.45	300	94.39	50	28.8	3.34	1,531.55	715.21	816.34
21/05/2019	M	97.5	4.52	817	100	92	26.1	9.96	1,486.63	671.6	815.03
12/4/2019	F	99	4.73	2,807	99.9	199	27.1	3.63	1,560.17	731	829.17
6/5/2019	M	102.2	5.2	380	98.44	84	27.3	16	1,841.95	823.95	1,018.00
6/5/2019	F	108	6.14	2,151	97.44	163	28.4	5.03	2,306.26	1,071.68	1,234.58
21/04/2019	F	113	7.03	2,948	100	184	28.2	2.04	2,433.18	1,160.48	1,272.70
20/07/2019	F	115	7.41	1,184	100	61	30.1	6.97	2,881.82	1,424.70	1,457.12
20/04/2019	F	121	8.63	1,548	100	148	28.9	16.11	3,255.34	1,458.90	1,796.44
12/4/2019	F	126	9.74	1,971	96.95	177	28.9	9.38	3,620.35	1,706.64	1,913.71
19/05/2019	M	127	9.97	1,311	100	147	28.9	19.17	3,925.03	1,786.53	2,138.50
6/4/2019	F	131	10.94	4,951	84.62	233	-	1.52†	-	-	-
6/5/2019	F	131.5	11.07	2,214	99.21	178	28.3	21.35	4,569.71	1,885.80	2,683.91
7/7/2019	F	133	11.45	1,744	99.87	124	28.4	14.42	4,310.59	1,990.40	2,320.19
8/5/2019	F	134.4	11.82	2,675	99.79	151	29.6	7.21	4,689.77	2,203.44	2,486.33
18/05/2019	M	144.5	14.68	2,361	99.89	173	28.7	10.63	6,080.72	2,673.90	3,406.82
6/4/2019	M	145	14.83	1,515	93.4	176	29.2	17.76	5,694.13	2,678.84	3,015.29
4/7/2019	F	154	17.76	1,374	96.71	112	28.7	16.71	7,358.18	3,382.10	3,976.08
5/5/2019	F	163	21.06	48	-	12	-	-‡	-‡	-‡	-‡
3/6/2019	M	179	27.87	13	-	1	-	-‡	-‡	-‡	-‡

Note: †Shark was removed from analysis due to an uncharacteristically small home range indicating that the shark died, or the tag was shed prematurely.
‡Insufficient data recorded for estimation of home range or metabolic rates.

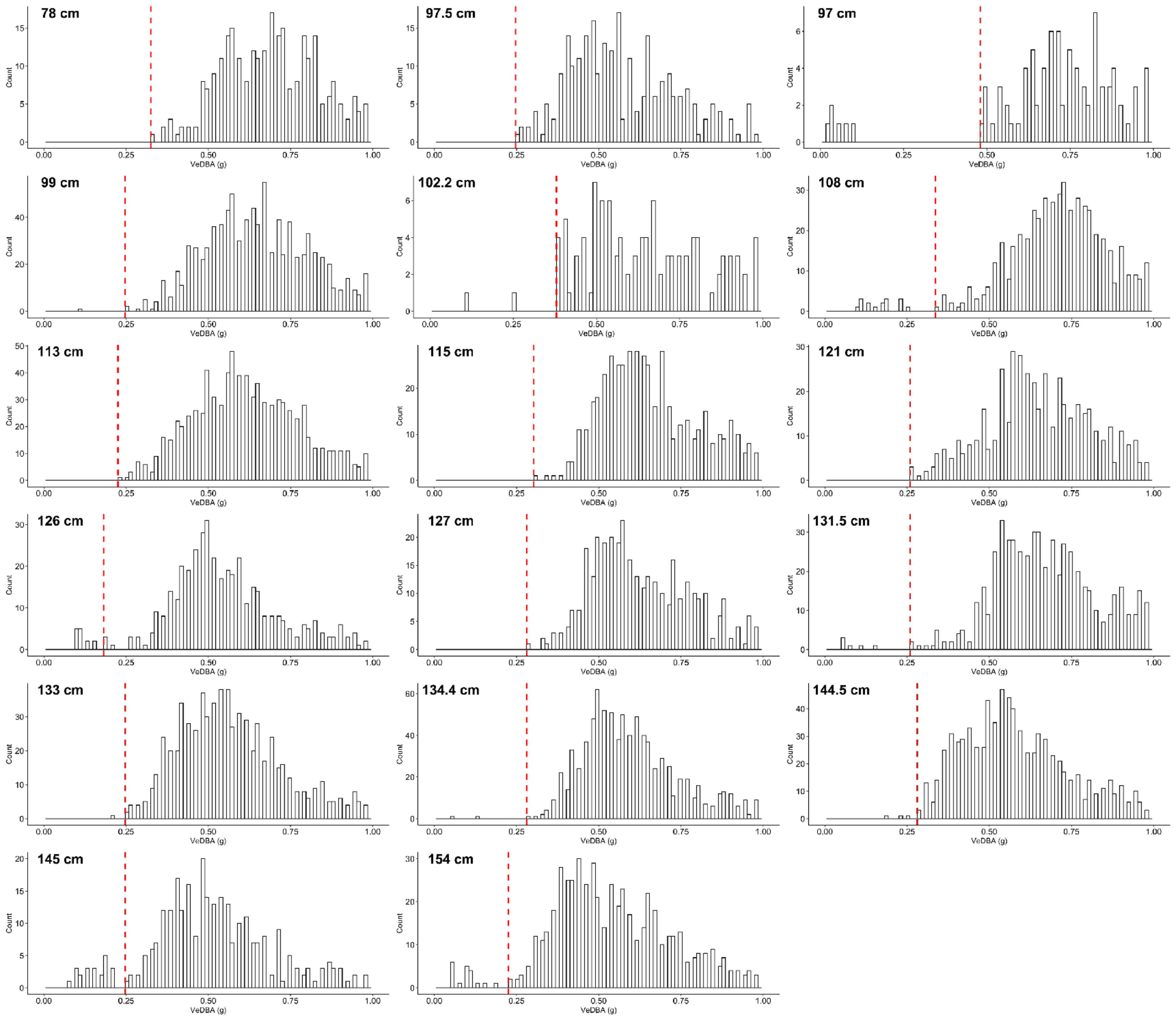

Figure S1. Histograms of vectorial dynamic body acceleration (VeDBA) for each shark used in analysis. Red dotted vertical line indicates the threshold value used to separate active from inactive VeDBA values for each shark, with values to the left of the line labelled as inactive and values to the right of the line labelled as active. A different threshold value was set for each individual and was determined by visually inspecting for a natural break in the histogram. This method was validated by observing similar patterns in acceleration data collected from sharks during respirometry experiments (personal observation).

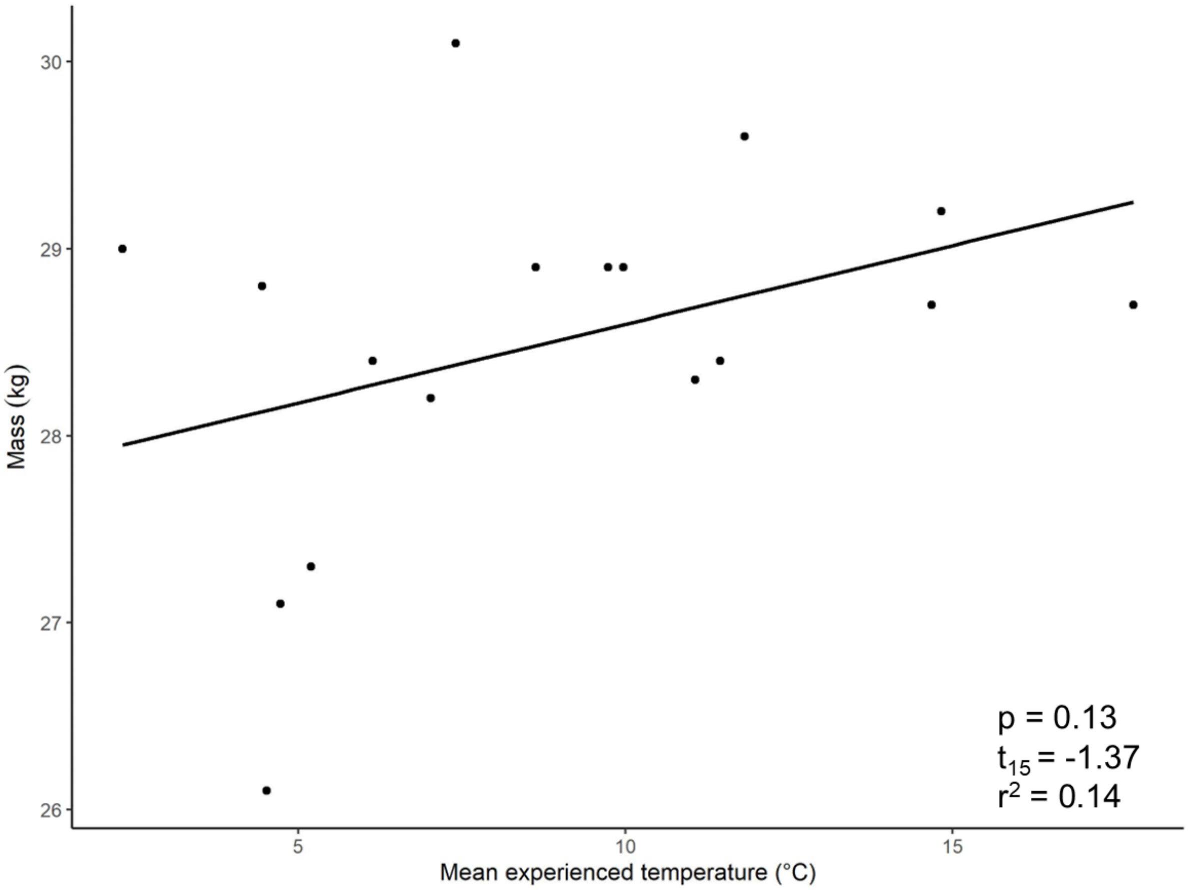

Figure S2. Mean daily temperature experienced by sharks plotted as a function of mass.

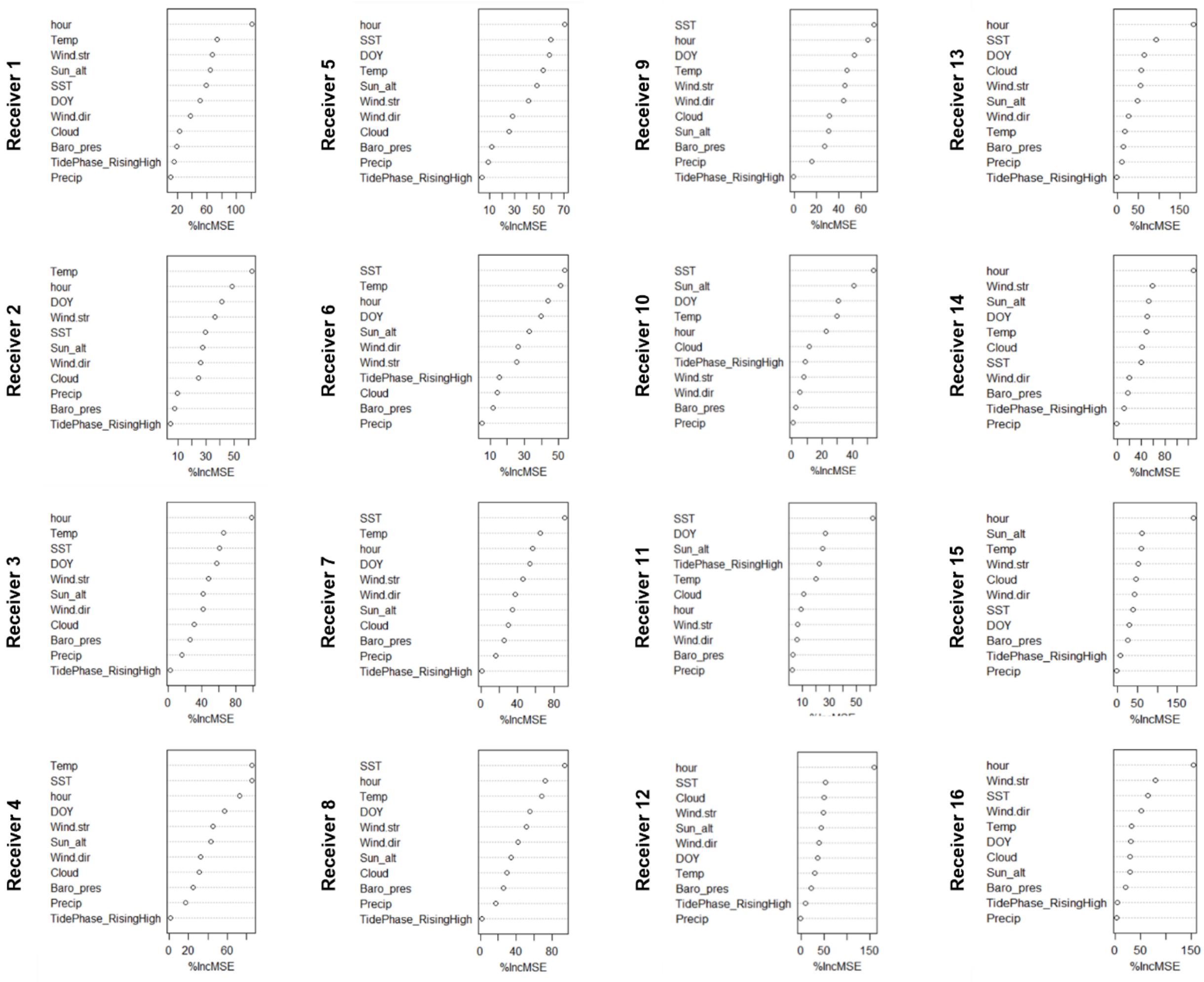

Figure S3. Variable importance within random forest models for all receivers. %IncMSE is the average increase in the model mean squared error when variables are randomly permutated, and is used as an indicator of variable importance. A higher %IncMSE value indicates greater change in model when variable is removed or added, therefore a higher value indicates higher variable importance. Models were fit to 16 to receivers with available temperature data.

References

Breiman, L. 2001. Random forests. Machine learning 45:5–32.

Gorman, B. 2018. mltools: Machine Learning Tools. CRAN, CRAN.

Kelley, D., and C. Richards. 2017. oce: Analysis of Oceanographic Data. R package version 0.9-22.

Lazaridis, E. 2014. lunar: Lunar Phase & Distance, Seasons and Other Environmental Factors. R package version 0.1-04.

Liaw, A., and M. Wiener. 2002. Classification and regression by randomForest. R news 2:18–22.

Naing, W. Y. N., and Z. Z. Htike. 2015. Forecasting of monthly temperature variations using random forests. ARPN journal of Engineering and Applied Sciences 10:10109–10112.
